# ApoE deficiency promotes colon inflammation and enhances the inflammatory potential of oxidized-LDL and TNF-α in primary colon epithelial cells

**DOI:** 10.1042/BSR20160195

**Published:** 2016-10-06

**Authors:** Ali H. El-Bahrawy, Abdelmetalab Tarhuni, Hogyoung Kim, Venkat Subramaniam, Ilyes Benslimane, Zakaria Y. Abd Elmajeed, Samuel C. Okpechi, Mohamed A. Ghonim, Ramadan A.M. Hemeida, Amira M. Abo-yousef, Gamal A. El-Sherbiny, Ihab T. Abdel-Raheem, Jong Kim, Amarjit S. Naura, A. Hamid Boulares

**Affiliations:** * The Stanley Scott Cancer Center, School of Medicine, Louisiana State University Health Sciences Center, New Orleans, LA 70112, U.S.A.; † Department of Pharmacology & Toxicology, Faculty of Pharmacy, Al-Azhar University, Assiut, 71524, Egypt; ‡ Department of Surgery, Tulane Medical Center, New Orleans, LA 70112, U.S.A.; § Department of Pharmacology & Toxicology, Beni-Suef University, Beni-Suef City, 62511, Egypt; ║ Department of Pharmacology & Toxicology, Kafrelsheikh University, Kafr El-Sheikh, 33516, Egypt; ¶ Department of Pharmacology & Toxicology Department, Damanhour University, Damanhour, 22111, Egypt

**Keywords:** Apolipoprotein E (ApoE), colon, cyclooxygenase (COX)-2, inflammation, NF-κB, oxidized-LDL

## Abstract

Although deficiency in Apolipoprotein E (ApoE) is linked to many diseases, its effect on colon homoeostasis remains unknown. ApoE appears to control inflammation by regulating nuclear factor-κB (NF-κB). The present study was designed to examine whether ApoE deficiency affects factors of colon integrity *in vivo* and given the likelihood that ApoE deficiency increases oxidized lipids and TNF-α, the present study also examined whether such deficiency enhances the inflammatory potential of oxidized-LDL (oxLDL) and TNF-α in colon epithelial cells (CECs), *in vitro*. Here we show that ApoE deficiency is associated with chronic inflammation systemically and in colonic tissues as assessed by TNF-α levels. Increased colon TNF-α mRNA coincided with a substantial increase in cyclooxygenase (COX)-2. ApoE deficiency enhanced the potential of oxLDL and TNF-α to induce COX-2 expression as well as several other inflammatory factors in primary CECs. Interestingly, oxLDL enhanced TGF-β expression only in ApoE^−/−^, but not in wild-type, epithelial cells. ApoE deficiency appears to promote COX-2 expression enhancement through a mechanism that involves persistent NF-κB nuclear localization and PI3 and p38 MAP kinases but independently of Src. In mice, ApoE deficiency promoted a moderate increase in crypt length, which was associated with opposing effects of an increase in cell proliferation and apoptosis at the bottom and top of the crypt respectively. Our results support the notion that ApoE plays a central role in colon homoeostasis and that ApoE deficiency may constitute a risk factor for colon pathologies.

## INTRODUCTION

The aetiology of many diseases can be linked to diet or changes in the contents of the diet. High fat diet and perturbation in cholesterol metabolism are increasingly regarded as determinants of numerous diseases including obesity, diabetes, hypertension and cancer with inflammation as a potential common denominator. Apolipoprotein E (ApoE) is a major player in cholesterol metabolism and reverse transport [1]. Deficiency in this lipoprotein has been linked to many vascular diseases including atherosclerosis as a result of an accumulation of total cholesterol and its oxidized form [2]. ApoE deficiency has also been shown to correlate with diseases of the liver [3], brain [4] and kidney [5]. ApoE appears to play a critical role in controlling inflammatory processes in these tissues through a control of key inflammation-driving factors such as nuclear factor-κB (NF-κB) and its signalling pathway [6]. Despite this knowledge, the role of ApoE in colon homoeostasis remains largely unknown. It is noteworthy that Wilson's group showed that an ApoE-mimetic peptide, termed COG112, attenuates colon inflammation in response to infection with *Citrobacter rodentium* or treatment with dextran sulfate sodium salt (DSS) in cells and animals with normal levels of ApoE [7]. As conducted, the present study did not address the actual role of ApoE in colon homoeostasis or the pathophysiological effects of ApoE deficiency but rather was more directed towards a correction of a preexisting inflammatory condition.

In humans, ApoE exists in three different isoforms expressed by a single gene locus with a difference residing at a couple of amino acids with a single residue substitution [8]. This difference governs the intensity by which the protein binds its receptor termed low-density lipoprotein (LDL) receptor-related protein-1 (LRP-1) [8]. Mice, however, express only one form of ApoE [8]. Through association studies, ApoE was suggested to play a role in colon homoeostasis and cancer with polymorphisms in the ApoE alleles constituting a risk factor for the development of adenoma and carcinoma of the colon [9,10]. A study by Kato et al. [11] challenged the notion that these polymorphisms predispose individuals to colon cancer. However, a recent study by Al-Meghaiseeb et al. [10] renewed the potential risk for colon cancer by associating ApoE polymorphisms with inflammatory bowel disease. These conflicting reports strongly call for a closer examination of the role of ApoE in the colon and the consequences of its deficiency. The normal architecture of the crypt is maintained by a delicate balance between cell proliferation at the base and apoptosis at the top of the crypt and on the surface epithelium. Alteration in this balance may lead to several pathologies ranging from chronic inflammation to cancer. The colon is often exposed to many components of dietary fats as well as enzymatic breakdown byproducts, which may influence the response of epithelial cells. Systemic increase in cholesterols may be associated with an increase in the oxidized form of LDL (oxLDL) [12] and cytokines such as tumour necrosis factor (TNF)-α and therefore cells from different tissues including colon epithelial cells (CECs) may also be exposed to such toxic byproducts and cytokines. Therefore, it is very likely that ApoE deficiency coexists with an increase in oxLDL. Accordingly, the present study was designed to examine whether ApoE deficiency affects colon integrity *in vivo* and determine whether it can enhance the inflammatory potential of oxLDL and TNF-α.

## MATERIALS AND METHODS

### Animals

Wild type (WT) and ApoE^−/−^ mice (*n* ≥ 6 per experimental group) were purchased from Jackson Laboratories. Mice were bred in a specific-pathogen free facility at LSUHSC and allowed unlimited access to sterilized chow and water. Maintenance and experimental protocols were all approved by the LSUHSC Animal Care & Use Committee.

### Organ recovery, tissue processing and assessments of sera for TNF-α and lipid contents

Mice were killed by carbon dioxide asphyxiation and blood was drawn by cardiac puncture. The colons were collected and prepared for primary CECs isolation, RNA or protein extraction or fixation with formalin as described [13]. Tissue sections were subjected to haematoxylin and eosin (H&E) staining or immunohistochemistry with antibodies specific to proliferating cell nuclear antigen (PCNA) (Santa Cruz Biotechnology) as described previously [13]. Specimens for RNA were immediately submerged in RNA later solution (Ambion). The measurement of lipid contents in sera of animals was done blindly by the Biomarker Core Laboratory (Emory University) as described [14]. TNF-α levels were measured in sera using a single-plex-based assay kit (RayBiotech). Colon crypt length was measured from the base to the tip of the crypt in crypts with a straight single layer of epithelial cells and with no disconnections. Similar criteria was used to count PCNA-positive cells in crypts and expressed as a percent number of positive cells of the total number of cells constituting the crypt. At least 40 crypts per mouse were counted.

### Isolation of primary colon epithelial cells, treatments and immunofluorescence

CECs were isolated essentially as described previously [13] with minor modifications. Briefly, colons were extracted from 6 weeks old mice and cleaned with cold calcium and magnesium-free Hanks balanced salt solution (HBSS) and then cut into smaller pieces for partial digestion with a cocktail of collagenase I (Sigma–Aldrich) and dispase (Thermo Fisher Scientific) in a complete Dulbecco's Modified Eagle's medium (DMEM) media. The generated crypts were subjected to differential attachment to yield fibroblast depleted crypts. Floating crypts were then placed on to petri dishes with a small amount of media to allow adherence. Cells were monitored for quality and used when they reach 80–90% confluency (∼10 days). Quality of CECs was monitored by immunofluorescence with pan-cytokeratin antibodies (Santa Cruz Biotech) and counterstained with DAPI as described [13]. Prior to treatment with oxLDL (Sigma–Aldrich) or TNF-α (Roche Applied Science), cells were starved for 5 h by incubation in serum-free medium containing 0.5% cell culture-tested BSA (Sigma–Aldrich). In some experiments, cells were pretreated with PP2, a proto-oncogene tyrosine-protein kinase (Src) inhibitor, LY294002 (Sigma–Aldrich), a pan-phosphoinositide (PI) 3-kinase (PI3K) inhibitor (Sigma–Aldrich), U0126 (Sigma–Aldrich), a mitogen-activated protein kinase (MAPK) kinase (MEK), SB203580, a p38 MAPK inhibitor, or SP600125, a c-Jun N-terminal kinase (JNK) inhibitor for 30 min. For immunofluorescence, cells grown on chamber slides were treated with oxLDL for different time intervals then fixed with 3.7% paraformaldehyde in PBS. Cells were permeabilized and subjected to immunofluorescence with antibodies to p65 NF-κB (Cell Signaling Technology; catalog# 8242) or pan-cytokeratin (Santa Cruz Biotech; catalog# sc-8018) followed by a counterstain with DAPI as described [15].

### Immunoblot analysis and conventional and quantitative RT-PCR

Protein extracts from cells or colon tissues were subjected to immunoblot analysis essentially as described [15] with antibodies to cyclooxygenase (COX)-2 (Cell Signaling Technology; catatog#4842S), cleaved (active) caspase-3 (Cell Signaling Technology; catatog#9664S), or actin (Santa Cruz Biotechnology; catatog#SC-8432). Immune complexes were detected with the appropriate secondary antibodies and chemiluminescence reagents (Thermo-Fisher Scientific). Blots were blindly quantified (by Dr Abd Elmageed or Dr Kim) using ImageJ (U.S. National Institutes of Health, Bethesda, MD, USA, http://imagej.nih.gov/ij/), normalized to actin and expressed as fold change from signals detected in extracts from control (unstimulated) cells or WT mice. Total RNA was extracted using the RNeasy Mini Kit (Qiagen) after which cDNA was generated using Reverse Transcriptase III (Invitrogen). The produced cDNA was subjected to conventional PCR using iQ supermix (Bio-Rad Laboratories) or subjected to quantitative PCR (QuantStudio™ Dx RT-PCR Ins., Applied Biosystem) using SYBR green RT-PCR master mix (Life Technologies) with primers (Supplementary Table S1) specific to mouse Vascular cell adhesion protein (VCAM)-1, COX-2, interleukin (IL)-1β, monocyte chemoattractant protein (MCP)-1, transforming growth factor (TGF)-β1, TNF-α or β-actin (IDT). Relative expression levels were calculated as described [16].

### Statistical analysis

All data are expressed as means ±S.D. of values from at least six mice per group. All experiments were conducted at least three times. PRISM software (GraphPad) was used to analyse the differences between experimental groups by one way ANOVA or unpaired *t* test.

## RESULTS

### ApoE deficiency causes chronic inflammation systemically and locally in the colon of mice

**Figure 1:**
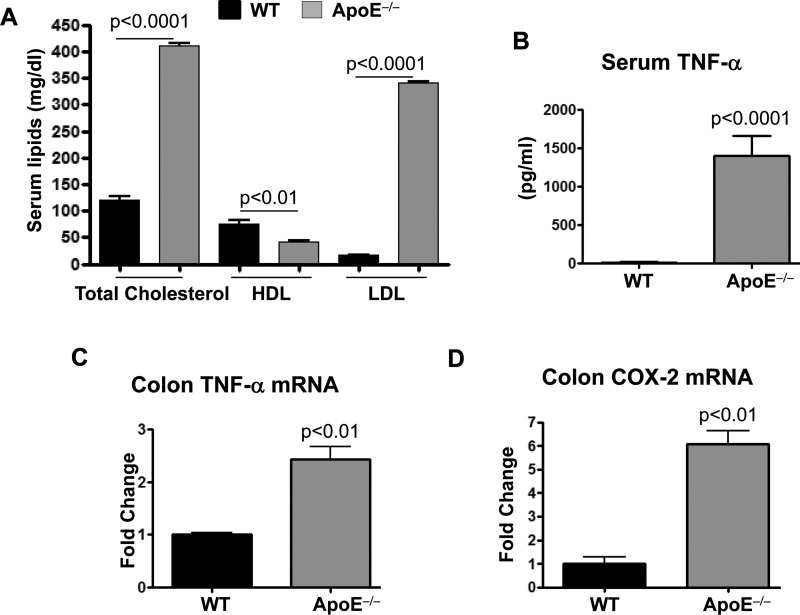
Effect of ApoE deficiency on systemic and colon inflammation in mice (**A**) Sera lipid profile (mg/dl) of WT and ApoE^−/−^ mice fed a regular diet. *, difference from WT mice. (**B**) Sera levels of TNF-α as assessed by a Bioplex assay. (**C** and **D**) Total RNA isolated from colon of WT or ApoE^−/−^ mice was subjected to reverse transcription followed by quantitative PCR with primers to mouse *TNF-α* (**C**), *COX-2* (**D**) or *β-actin* and is expressed as fold change over levels detected in WT tissues. *, difference from WT mice.

Since a mere deficiency in ApoE increases total cholesterol and LDL levels without the addition of a high fat diet regimen (Figure 1A), we wished to examine whether ApoE deficiency was sufficient to increase TNF-α levels in sera of animals fed a diet with normal levels of cholesterol. Figure 1(B) shows that, indeed, ApoE gene deletion promoted systemic inflammation as demonstrated by the ∼100-fold increase in sera TNF-α levels of mice that received regular diet for 16 weeks over those detected in sera of the WT counterparts.

We previously showed that high fat diet-fed ApoE^−/−^ mice display elevated levels of TNF-α in the lungs [17], suggesting a role in systemic as well as local inflammation [17]. This led us to speculate that other tissues such as the colon may also be affected. Figure 1(C) shows that TNF-α mRNA levels in the colon of ApoE^−/−^ mice were more than two folds higher than those detected in colon of WT mice under the same regular diet regimen. We next examined whether the increased TNF-α expression correlated with an increase in the expression of the pro-inflammatory enzyme COX-2. Figure 1(D) shows that COX-2 expression was approximately six times higher in colon of ApoE^−/−^ mice compared with the WT counterpart. Altogether, these results suggest that ApoE deficiency and perhaps the associated hypercholesterolemia are critical for colon homoeostasis.

### ApoE deficiency enhances the potential of oxLDL to induce expression of COX-2 as well as MCP-1, IL-1β, ICAM-1, VCAM-1 and TGF-β in primary colon epithelial cells

**Figure 2:**
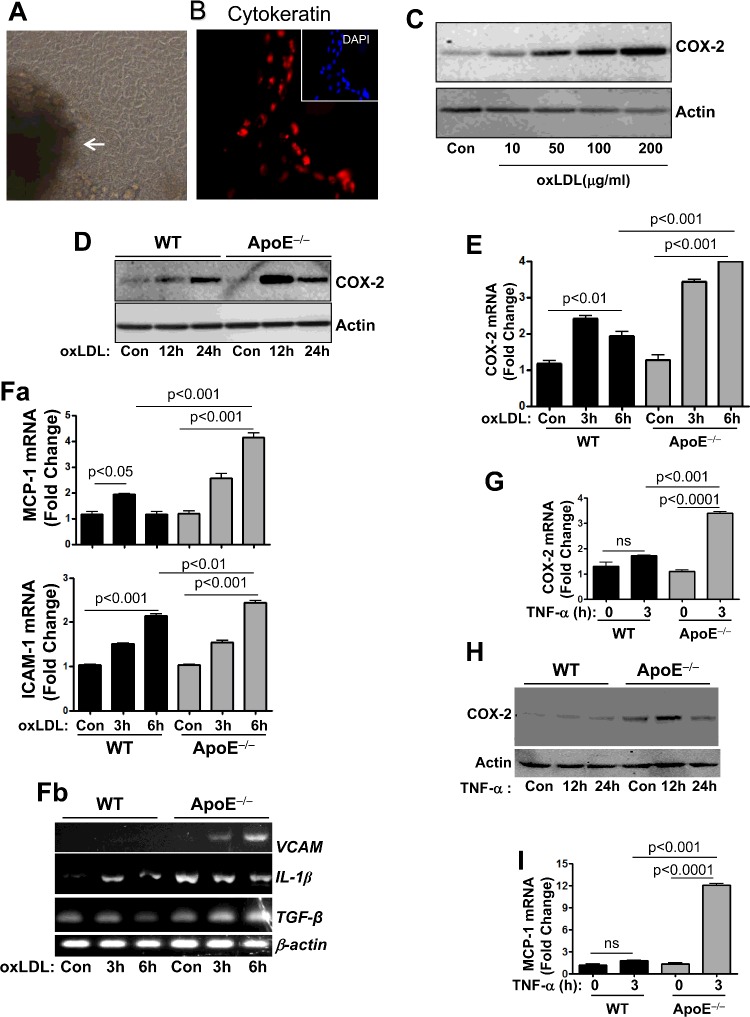
ApoE deficiency enhances the potential of oxLDL to induce expression of COX-2 and other inflammatory and growth modulatory factors CECs prepared from colon of WT or ApoE^−/−^ mice using a differential attachment method. (**A**) Cells were visualized by bright field microscopy; white arrow indicates the colonic crypt from which the cells originated. (**B**) Cells cultured in chamber slides were fixed with formalin and subjected to immunofluorescence with antibodies to pan-cytokeratin (red) or staining with DAPI (blue; insert). (**C**) CECs isolated from WT mice were treated with 10, 50, 100 or 200 μg/ml oxLDL for 24 h. Protein extracts were subjected to immunoblot analysis with antibodies to mouse COX-2 or actin. (**D**) WT or ApoE^−/−^ CECs were treated with 100 μg/ml oxLDL for 12 or 24 h. Protein extracts were subjected to immunoblot analysis with antibodies to mouse COX-2 or actin. (**E**) WT or ApoE^−/−^ CECs were treated with 100 μg/ml oxLDL for 3 or 6 h. Total RNA was reversed transcribed and the resulting cDNA was amplified by quantitative PCR with primers specific to mouse *COX-2* or *β-actin*. (**Fa** and **Fb**) Cells were treated as in (**E**) and the cDNA generated from the isolated RNA was amplified by quantitative PCR (**Fa**) with primers to mouse *MCP-1 or ICAM-1 or by conventional PCR* (**Fb**) with primers to *IL-1β, VCAM-1, TGF-β or β-actin.* (**G**) WT or ApoE^−/−^ CECs were treated with 10 ng/ml TNF-α for 3 h. cDNA generated from the isolated RNA was amplified by quantitative PCR with primers to mouse *COX-2* or *β-actin*. (**H**) WT or ApoE^−/−^ CECs were treated with 10 ng/ml TNF-α for 12 or 24 h. Protein extracts were subjected to immunoblot analysis with antibodies to mouse COX-2 or actin. (**I**) The same cDNA from (**G**) was amplified by quantitative PCR with primers to mouse *MCP-1* or *β-actin*.

An accumulation in LDL upon ApoE gene depletion was shown to result in increased levels of oxLDL [18], which is thought to be an important mediator of inflammation systemically as well as outside the vasculature. We therefore wished to examine whether ApoE deficiency modifies CEC responses to oxLDL. To conduct this experiment, we utilized primary CECs that were isolated using a methodology developed by our laboratory [19]. Figure 2(A) shows CECs stemming from a colonic crypt. The epithelial nature of the isolated cells was assessed by immunofluorescence with antibodies to pan-cytokeratin (Figure 2B). After 7–10 days in culture, CECs were serum starved for 5 h prior to treatment with different concentrations of oxLDL (10, 50, 100 or 200 μg/ml) for 24 h to determine their sensitivity to the oxidized lipid. Cells were collected and subjected to protein extraction followed by immunoblot analysis with antibodies to mouse COX-2. Figure 2(C) shows that oxLDL efficiently induced expression of COX-2 in a dose dependent manner. The 100 μg/ml concentration of oxLDL, which is widely used in cardiovascular research, was selected to conduct the studies.

CECs, isolated from age-matched WT or ApoE^−/−^ mice, were serum starved prior to treatment with 100 μg/ml oxLDL for 12 or 24 h. Protein extracts from the collected cells were subjected to immunoblot analysis with antibodies to mouse COX-2. Figure 2(D) shows that ApoE deficiency not only promoted a marked increase in COX-2 protein expression compared with those in similarly treated WT cells, but also accelerated the time of expression. Figure 2(E) shows that the enhancement of oxLDL-induced COX-2 protein expression as a result of ApoE deficiency appeared to take place at the mRNA level. The potentiation of ApoE deficiency of the effect of oxLDL was not limited to COX-2 but also extended to other inflammatory factors including MCP-1, IL-1β, ICAM-1 and VCAM-1 (Figures 2Fa and 2Fb). It is noteworthy that ApoE deficiency was also associated with increased expression of the basal levels of IL-1β independently of oxLDL exposure (Figure 2Fb). Increases in COX-2 levels as well as the other tested factors were inconsistently observed in untreated control ApoE^−/−^ CECs (results not shown). Although oxLDL was not efficient in inducing VCAM-1 expression in WT CECs, it promoted a time-dependent expression of the adhesion molecule in ApoE^−/−^ CECs (Figure 2Fb). Interestingly, oxLDL enhanced expression of TGF-β only in ApoE^−/−^ CECs but not in the WT cells (Figure 2Fb). Altogether, these results support the notion that ApoE may play an important role in preventing an inflammatory environment in the colon in response to elevated levels of oxLDL.

Given that ApoE deficiency is associated with increased TNF-α expression in the colon, it thus became plausible to predict that such deficiency would enhance the potency of TNF-α in inducing COX-2 expression. It is important to mention that the effect of ApoE deficiency on TNF-α-induced factors in the colon or CECs has never been examined. To test the effect of ApoE deficiency on the efficacy of TNF-α to induce COX-2 and other factors in CECs, cells isolated from WT or ApoE^−/−^ mice were treated with 10 ng/ml mouse TNF-α. Figures 2(G) and 2(H) show that although the concentration of TNF-α used in this experiment was inefficient in inducing detectable amounts of COX-2 mRNA or protein expression in CECs derived from WT mice, it induced substantial amounts of the COX mRNA and protein in ApoE^−/−^ CECs. The 10 ng/ml TNF-α concentration was very efficient at inducing COX-2 in the airway epithelial cell line A549 as well (Supplementary Figure S1) suggesting that CECs are relatively resistant to stimulation by TNF-α. Similarly to COX-2, TNF-α at the 10 ng/ml concentration did not induce expression of MCP-1 in WT CECs; however, it was very efficient at inducing this chemokine in ApoE^−/−^ CECs (Figure 2I). Overall, these results suggest that ApoE deficiency sensitizes CECs to TNF-α stimulation as well as to oxLDL.

### ApoE deficiency enhances oxLDL-induced COX-2 expression potentially through a mechanism that involves persistent NF-κB nuclear localization, PI3 kinases and p38 MAPK, but independently of Src

**Figure 3:**
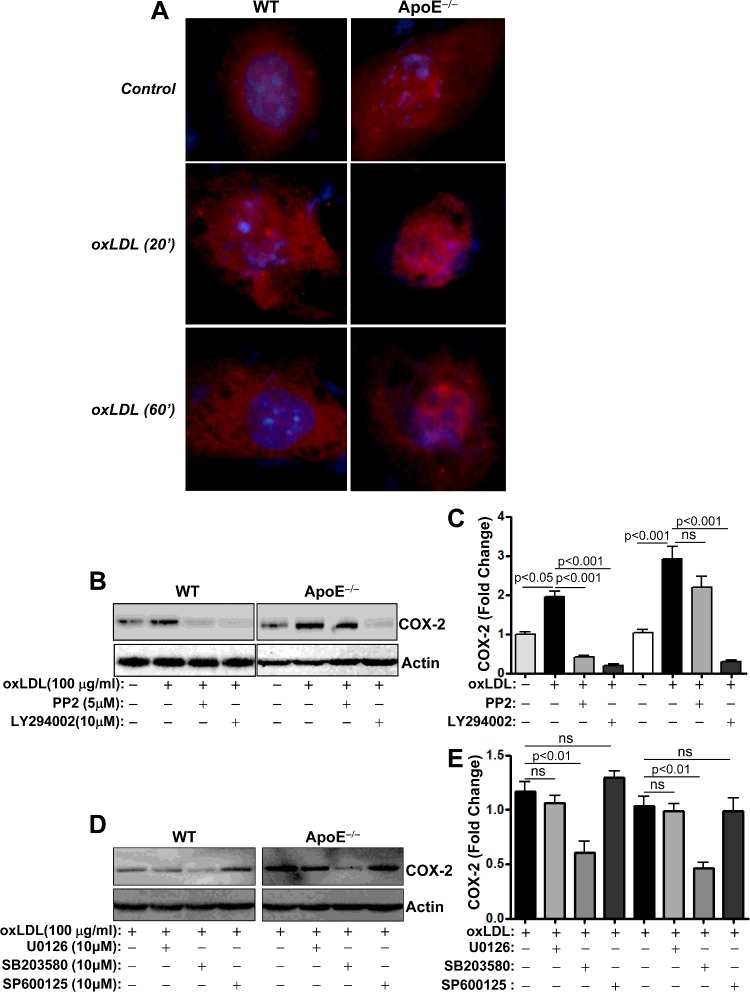
Effects of ApoE deficiency on oxLDL-induced NF-κB nuclear translocation and sensitivity of COX-2 protein expression to Src. PI3K, MEK, p38 MAPK or JNK inhibition (**A**) WT or ApoE^−/−^ CECs cultured in chamber slides were treated with 100 μg/ml oxLDL for 20 or 60 min. Cells were then fixed with formalin and subjected to immunofluorescence with antibodies to p65 NF-κB and counterstained with DAPI. (**B**) WT or ApoE^−/−^ CECs were pretreated with the Src inhibitor PP2 (5 μM) or the PI3K inhibitor LY294002 (10 μM) prior to treatment with 100 μg/ml oxLDL for 24 h. Protein extracts were subjected to immunoblot analysis with antibodies to COX-2 or actin. (**C**) The blots in (B) were quantified using ImageJ and normalized to actin levels. (**D**) WT or ApoE^−/−^ CECs were treated with the MEK inhibitor U0126, the p38MAPK inhibitor SB203580 or the JNK inhibitor SP600125 prior to treatment with oxLDL. Protein extracts were subjected to immunoblot analysis with antibodies to mouse COX-2 or actin. (**E**) The blots in (D) were quantified using ImageJ and normalized to actin levels.

NF-κB is considered as a key regulator of mucosal inflammation and is known to mediate oxLDL-induced signalling to drive target genes [20]. Furthermore, NF-κB signalling can be modulated by ApoE [6]. It is noteworthy that the genes examined in that study are known targets of NF-κB. Accordingly, it became important to examine whether oxLDL enhances NF-κB signalling in ApoE^−/−^ CECs. To this end, we examined the effect of oxLDL and ApoE deficiency on the nuclear translocation of NF-κB by immunofluorescence using antibodies to the p65 subunit of the transcription factor. Figure 3(A) shows that even in the absence of oxLDL and upon serum starvation, some nuclear p65 NF-κB was already apparent in ApoE^−/−^ CECs, which was completely absent from WT cells. Although treatment with oxLDL induced a transient p65 NF-κB nuclear localization in WT CECs, such features seemed to be more persistent in oxLDL-treated ApoE^−/−^ cells. These results may be, in part, the driving force behind the sensitivity of ApoE^−/−^ cells to oxLDL and the enhanced expression of inflammatory genes.

Very recently oxLDL was reported to influence COX-2 expression, in part, through the activity of the proto-oncogene non-receptor tyrosine-protein kinase Src and PI3K [21]. Figures 3(B) and 3(C) show that, indeed, oxLDL-induced expression in WT CECs was highly sensitive to PP2 treatment, a Src inhibitor, and to LY294002, a pan-PI3K inhibitor. Surprisingly however, although oxLDL-induced COX-2 expression was sensitive to PI3K inhibition, its sensitivity to Src inhibition was minimal. Figures 3(D) and 3(E) show that COX-2 expression in oxLDL-treated WT and ApoE^−/−^ CECs was equally sensitive to p38 MAP kinase inhibition by SB203580 or resistant to JNK inhibition by SP600125. Additionally, ApoE deficiency did not influence the partial relative sensitivity of oxLDL-induced COX-2 expression to MEK inhibition. These results suggest that ApoE deficiency may promote induction of COX-2 expression in CECs through a PI3K-dependent, but Src-independent, mechanism.

### ApoE deficiency promotes a moderate increase in crypt length, which may be associated with opposing effects of an increase in cell proliferation and apoptosis

**Figure 4:**
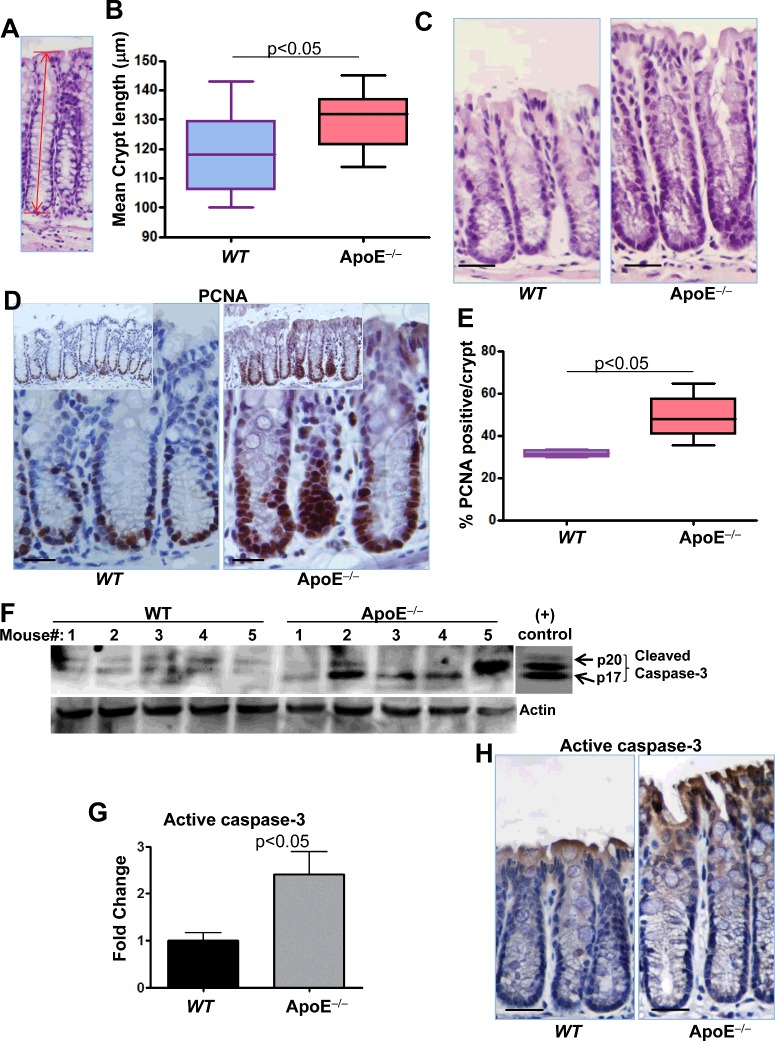
Effect of ApoE deficiency on crypt length, cell proliferation and apoptosis Colonic sections from WT or ApoE^−/−^ mice were stained with H&E and crypts length was measured as displayed in (**A**). (**B**) Average of at least 40 crypts per colon in each group. (**C**) Representative pictures of WT and ApoE^−/−^ colonic tissues. (**D**) Colonic sections from WT or ApoE^−/−^ mice were subjected to immunohistochemistry with antibodies to PCNA. (**E**) PCNA-positive cells were counted along the crypts and expressed as percent of total cells per crypt. (**F**) Protein extracts prepared from colons of WT or ApoE^−/−^ mice were subjected to immunoblot analysis with antibodies to the active form of caspase-3 (p17 and p20) or actin; the rightmost panel represents immunoblot of purified caspase-3 as a positive control. (**G**) The immunoblots were quantified using ImageJ and normalized to actin levels. Colonic sections from WT or ApoE^−/−^ mice were subjected to immunohistochemistry with antibodies to active caspase-3. Bars in C, D and H=30 μm.

Changes in the homoeostasis of the colon are often reflected by changes in the length of the colon and its crypts [22]. We thus examined whether the pro-inflammatory conditions locally in the colon and systemically in ApoE^−/−^ mice affected colon homoeostasis. A gross examination of the colons revealed no obvious difference in the overall structure or length of the organ between WT and ApoE^−/−^ mice that received a regular diet regimen for 16 weeks (results not shown). However, microscopic measurements, as shown in Figure 4(A), revealed that the length of crypts was moderately increased in colonic tissues of ApoE^−/−^ mice compared with the WT counterparts (Figures 4B and 4C). It is important to note that such difference reached statistical significance (*P*=0.0263). To determine whether the increase in colonic crypt length of ApoE^−/−^ mice was associated with an increase in proliferation, we assessed the tissues by immunohistochemistry with antibodies to PCNA, a marker of proliferation. Figures 4(D) and 4(E) show that prevalence of nuclear PCNA was significantly higher in colonic crypts of ApoE^−/−^ mice compared with those of WT mice. These results suggest that proliferation of CECs in ApoE^−/−^ mice was higher than those of WT mice.

Given that apoptosis plays a critical role in maintaining homoeostasis of the colonic mucosa, we examined whether ApoE deficiency was associated with changes in the apoptotic response. To this end, colons from WT or ApoE^−/−^ mice were subjected to protein extraction followed by immunoblot analysis with antibodies to the active form of caspase-3, a major apoptotic enzyme. Figures 4(F) and 4(G) show that ApoE deficiency was associated with an increase in the levels of active caspase-3 compared with those of the WT. It appears that the increase in caspase-3-immunoreactivity is at the top of the crypt with no evidence of caspase-3 activation within PCNA-positive cells (Figure 4H). The above results suggest that ApoE plays an important role in maintaining the homoeostasis of the colon and that deficiency in this protein may compromise the integrity of the tissue.

## DISCUSSION

ApoE is increasingly regarded as an important player in the homoeostasis of a number of tissues including the kidney [5], the liver [3] and the brain [4]. The results of our study demonstrate that the lipoprotein also plays an important role in colon homoeostasis and that its deficiency may render the tissue environment pro-inflammatory. ApoE deficiency appears to increase the potency of oxLDL and TNF-α in inducing the expression of inflammatory factors such as COX-2 as well as cytokines, chemokines and adhesion molecules, in part, through an enhancement of the NF-κB pathway. Interestingly, the requirement of Src for oxLDL-induced COX-2 expression seems to be bypassed upon ApoE deficiency. ApoE deficiency was also associated with an increase in crypt length but with opposing effects on cell fate; both proliferation and apoptosis were increased. This increased inflammation and perturbation in cell fate may be conducive to more serious consequences if additional negative conditions present themselves within the tissue leading, perhaps, to maladies such as colitis and colon cancer. Indeed, inflammation is a critical driving force in tumorigenesis and pre-existing inflammatory conditions dramatically increases the risk of cancer development at the inflammatory site [23]. Overall, our study calls for caution about the effects of ApoE deficiency not only in lipid metabolism and the subsequent risk for hypercholesterolemia but also as a major risk factor for diseases of the colon.

The role of COX-2 in inflammation of the colon is well established [24]. A number of pathologies including colitis and colon cancer are linked to an overexpression of the inducible COX [24]. Furthermore, inhibitors of COX-2 such as rofecoxib ameliorate irritants-induced colitis in a mouse model of the condition [25]. The relationship between COX-2 and ApoE deficiency is not new as COX-2 levels were shown to be elevated in renal tissues from naïve mice [26]. However, the observation that ApoE deficiency enhances expression of the enzyme in response to oxLDL or TNF-α in CECs is novel. Deficiency in ApoE and other lipid metabolism-associated proteins are often associated with an increase in serum LDL levels with a subsequent increase in oxLDL [18]. Accordingly, the potentiation of oxLDL-induced inflammation is very relevant not only for the colon but also to many other tissues and organs. In fact, ApoE^−/−^ mice display an age-dependent increase in oxLDL [18]. We believe that the level of inflammation caused by ApoE deficiency may be moderate and does not cause obvious pathologies in the 16-week time frame examined in that study. However, it may provide conditions conducive to the establishment of pathologies if additional conditions present themselves. For instance, although ApoE^−/−^ mice develop atherosclerosis in the absence of a high fat diet, this condition is substantially accelerated when mice are fed a cholesterol-rich diet [14]. Recently, Levine and co-workers showed that asthma-like traits are enhanced in ApoE^−/−^ mice when exposed to house dust mite extracts in the form of an allergen [27]. Furthermore, hypercholesterolaemia associated with ApoE deficiency was shown to lead to renal injury [5].

Inflammatory conditions are commonly associated with the activation of several inflammatory transcription factors, including NF-κB. Activation of NF-κB not only promotes inflammation and expression of inflammatory genes, it can also promote enhancement and subsequent persistence of inflammation. The connection between ApoE and NF-κB has been previously described [6]. Our findings confirm such relationship in the colon and show that ApoE deficiency enhances the subcellular localization of the transcription factor even in the absence of any stimuli. Such enhanced and persistent nuclear localization may be, in part, the mechanism by which ApoE deficiency enhances the expression of target genes in response to oxLDL treatment including COX-2, ICAM-1, MCP-1 and TNF-α. Li et al. [28] showed that COG112, an ApoE-mimetic peptide, blocks production of TNF-α and IL-6 in peritoneal macrophages treated with a combination of LPS and IFN-γ. In a caecal ligation and puncture sepsis model, Wang et al. [29] showed that the ApoE-mimetic peptide COG1410 reduced TNF-α, IL-1β, IL-6 and IL-12 levels in mice expressing either the human ApoE3 or ApoE4 isoform of the lipoprotein. In brain pathologies, ApoE deficiency was shown to lead to blood–brain barrier breakdown after injury [30]. Interestingly, although ApoE2 and ApoE3 appear to be protective, ApoE4 enhances such injury [31] and subsequent production of inflammatory factors such as cyclophilin A and MMP-9 [32]. Although the mechanism by which ApoE influences NF-κB is not clear, a few suggestions have been made. The ApoE-mimetic peptide COG112 was shown to block production of many inflammatory factors including TNF-α, KC, IL-17 and MIP-2 potentially by preventing nuclear accumulation of NF-κB and activation of IKK in CECs in a model of colitis [7]. In blood–brain barrier breakdown-associated inflammation, ApoE2 and ApoE3 were suggested to bind to LRP-1 resulting in suppression of NF-κB by blocking the cyclophilin A–MMP-9 pathway [32]. Ironically, ApoE4 appeared to enhance such pathway as a result of its failure to efficiently bind LRP-1 [32]. However, ApoE deficiency-associated increase in MCP-1 and COX-2 in the kidney was reversed by an ApoA-1 mimetic peptide by reducing nuclear accumulation of NF-κB through a mechanism that involves dampening of IKK activity as assessed by the levels of IκBα phosphorylation [26]. Furthermore, an additional level of complexity has recently been reported on the mechanism by which ApoE regulates NF-κB which entails an enhancement of microRNA-146a [33].

Expression of COX-2 in response to oxLDL [26] and expression of TNF-α in response to LPS [34] can be regulated by Src. Our results, although preliminary, show that ApoE deficiency bypasses the requirement for Src, which may be evidence for an additional mechanism by which ApoE regulates expression of COX-2 and perhaps other NF-κB-dependent genes. Overall, these aforementioned reports and the findings of our study exemplify the complexity by which ApoE (and its isoforms) controls inflammation and mediates tissue homoeostasis.

ApoE deficiency was also associated with an increase in colonic crypt length. This result was unexpected given the fact that chronic inflammation often is associated with a shortening of the crypts due to the increase in cell turnover. Such increase in length was most probably due to an increase in cell proliferation as assessed by the percent of PCNA-positive epithelial cells. Consistent with these results, ApoE deficiency was also associated with a slight increase in PCNA-positive cells in the kidney injury model reported by Wen et al. [5]. It is noteworthy that the modest increase in crypt length could not explain the significant increase in PCNA-positive CECs and thus, an induction of apoptosis was expected. According to our immunohistochemistry results, an increase in immunoreactivity of active caspase-3 as an indicator of apoptosis was observed at the top of the colonic crypt with no evidence of caspase-3 activation within PCNA-positive cells. The coexistence of these two opposing effects is contradictory and is difficult to explain. However the induction of apoptosis in ApoE deficient tissues is not without a precedent. Indeed, ApoE was recently shown to protect astrocytes from hypoxia-induced apoptosis [4]. It is tempting to speculate that the increase in apoptosis may be due to the observed increase in TGF-β. TGF-β is known to act through its signalling pathway to arrest the cell cycle at the G1 stage to induce differentiation or promote apoptosis in the colon. Overall, our results suggest an important role for ApoE in colon homoeostasis and integrity and predict that ApoE deficiency may be a risk factor for chronic diseases such as colitis and colon cancer. Obviously, a great deal of work is necessary to test these predictions.

## Abbreviations

ApoE: Apolipoprotein E
COX: cyclooxygenase
CEC: colon epithelial cell
H&E: haematoxylin and eosin
IL: interleukin
JNK: c-Jun N-terminal kinase
LDL: low-density lipoprotein
LRP-1: receptor-related protein-1
NF-κB: nuclear factor-κB
oxLDL: oxidized-LDL
PCNA: proliferating cell nuclear antigen
PI3K: phosphoinositide 3-kinase
