# Correction: ApoE deficiency promotes colon inflammation and enhances the inflammatory potential of oxidized-LDL and TNF-α in primary colon epithelial cells

**DOI:** 10.1042/BSR20160195COR

**Published:** 2016-11-17

**Authors:** Ali H. El-Bahrawy, Abdelmetalab Tarhuni, Hogyoung Kim, Venkat Subramaniam, Ilyes Benslimane, Zakaria Y. Abd Elmageed, Samuel C. Okpechi, Mohamed A. Ghonim, Ramadan A.M. Hemeida, Amira M. Abo-yousef, Gamal A. El-Sherbiny, Ihab T. Abdel-Raheem, Jong Kim, Amarjit S. Naura, A. Hamid Boulares

Volume 36, issue 5 (2015) e00388; DOI: 10.1042/BSR20160195

The name of the author Zakaria Y. Abd Elmageed was spelled incorrectly (as Zakaria Y. Abd Elmajeed) in the published article. The correct spelling appears here.

